# Recent advances in understanding telomere diseases

**DOI:** 10.12703/r/11-31

**Published:** 2022-10-19

**Authors:** Vinicius S Carvalho, Willian R Gomes, Rodrigo T Calado

**Affiliations:** 1 Department of Medical Imaging, Hematology, and Oncology, Ribeirão Preto Medical School, University of São Paulo, Ribeirão Preto, São Paulo, Brazil

**Keywords:** telomere, telomerase, aplastic anemia, pulmonary fibrosis, liver cirrhosis, dyskeratosis congenita, myeloid neoplasm

## Abstract

Germline genetic defects impairing telomere length maintenance may result in severe medical conditions in humans, from aplastic anemia and myeloid neoplasms to interstitial lung disease and liver cirrhosis, from childhood (dyskeratosis congenita) to old age (pulmonary fibrosis). The molecular mechanisms underlying these clinically distinct disorders are pathologically excessive telomere erosion, limiting cell proliferation and differentiation, tissue regeneration, and increasing genomic instability. Recent findings also indicate that telomere shortening imbalances stem cell fate and is associated with an abnormal inflammatory response and the senescent-associated secretory phenotype. Bone marrow failure is the most common phenotype in patients with telomere diseases. Pulmonary fibrosis is a typical phenotype in older patients, and disease progression appears faster than in pulmonary fibrosis not associated with telomeropathies. Liver cirrhosis may present in isolation or in combination with other phenotypes. Diagnosis is based on clinical suspicion and may be confirmed by telomere length measurement and genetic testing. Next-generation sequencing (NGS) techniques have improved genetic testing; today, at least 16 genes have been implicated in telomeropathies. NGS also allows tracking of clonal hematopoiesis and malignant transformation. Patients with telomere diseases are at high risk of developing cancers, including myeloid neoplasms and head and neck cancer. However, treatment options are still limited. Transplant modalities (bone marrow, lung, and liver) may be definitive to the respective organ involvement but limited by donor availability, comorbidities, and impact on other affected organs. In clinical trials, androgens elongate telomeres of peripheral blood leukocytes and improve hematopoiesis. Further understanding of how telomere erosion impairs organ function and how somatic mutations evolve in the hematopoietic tissue may help develop new strategies to treat and prevent telomere diseases.

## Introduction

### What are telomeres and telomerase?

Telomeres are repetitive hexanucleotide (5′-TTAGGG-3′) sequences at the ends of linear chromosomes whose function is to maintain the cell genomic integrity and prevent the natural chromosome ends from being recognized as damaged DNA by the DNA repair machinery^1,2^. Telomeres are covered by a six-protein complex called shelterin (TRF1, TRF2, TIN2, TPP1, RAP1, and POT1) that binds directly or indirectly to the telomeric DNA for protection and to form a lariat structure (the “t-loop”)^3^. Shelterin protects telomeric sequences and regulates the access of other enzymes, such as telomerase and helicases, to telomeric DNA^4^. Without this complex telomeric structure, the 3′ and 5′ ends of linear chromosomes could be recognized as pathological DNA breaks, engaging DNA repair, and thus abnormally provoking chromosome end-to-end fusions.

Telomeres shorten at each mitotic cell division, and critically short telomeres trigger DNA damage responses, engaging cell senescence and apoptosis^5^. Recent findings, however, alternatively suggest that short telomeres divert stem cell division towards differentiation at the expense of self-renewal, thus exhausting the stem cell pool, at least in the hematopoietic compartment^6^. Skewed hematopoietic differentiation capacity also occurs when differentiating induced pluripotent stem cells derived from patients with mutations^6,7^. Cells with short telomeres display impaired endothelial-to-hematopoietic transition, and definitive hematopoietic accumulation is perturbed by DNA accumulation signaled via p53^8^. Imbalanced stem cell fate also may recruit quiescent cells bearing deleterious somatic mutations and favor clonal hematopoiesis. These recent findings expand how we understand the mechanisms by which telomere attrition limits cell proliferation, possibly by inducing senescence or apoptosis and modifying the stem cell fate.

**Figure 1.**
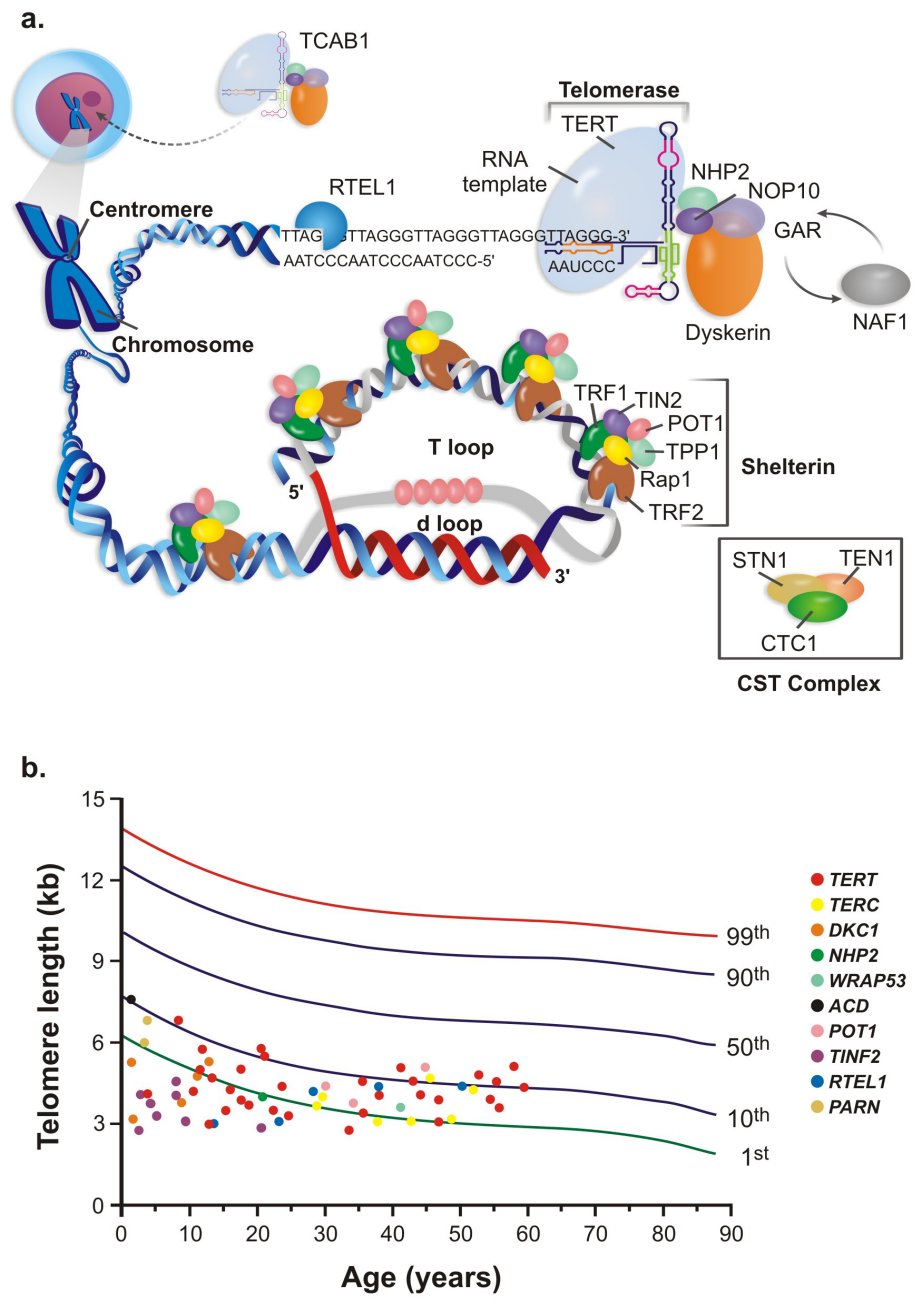
Telomeres and telomerase. (**A**) The telomerase complex is composed of the reverse transcriptase telomerase, the RNA template TERC, and associated proteins. It adds hexameric repeats to the 3′ end of the leading DNA telomeric strand. (**B**) Percentile distribution and telomere length (kilobases) of peripheral blood leukocytes in patients harboring mutations in telomere-related genes. Telomeres are longer at birth and gradually shorten with aging. TERC, telomerase RNA component.

On the other hand, highly proliferative cells, such as cancer and embryonic stem cells, maintain their telomeres by the action of telomerase, a reverse transcriptase that adds telomeric nucleotide sequences at the 3′ end of the DNA leading strand using an RNA molecule as a template. The telomerase complex includes the reverse transcriptase enzyme (TERT), an RNA component (TERC) that provides the template for telomere elongation, and the stabilizing proteins (dyskerin, NHP2, GAR1, NOP10, and NAF1), which participate in the assembly and stabilization of TERC, optimizing telomerase activity and processivity (Figure 1)^9^. More recently, other proteins have been directly or indirectly implicated in telomere maintenance. The helicase RTEL1 unfolds the telomeric structure and resolves G-quadruplex structures, allowing telomerase to access DNA and lengthen telomeres^10^. PARN is an RNase responsible for TERC deadenylation, thus preventing degradation^11^. TCAB1 is responsible for the trafficking of telomerase from the Cajal bodies into the nucleolus, where telomeres are elongated^12,13^. The CST complex (STN1, TEN1, and CTC1) is a telomerase regulator, working as a switch to restrict telomere elongation to one event per cell cycle^14^.

Although telomeres were initially thought to be transcriptionally inert, they produce a complex group of long non-coding RNAs termed TERRA (telomeric repeat-containing RNA)^15^. TERRA appears to participate in telomere protection, replication, and elongation^16^. In humans, TERRA transcription is regulated by the TERRA promoter methylation, as most human distal subtelomeric regions are enriched with CpG islands. TERRA transcription is tightly controlled, and its dysregulation may result in telomere erosion. Reduced TERRA transcription may result in telomere DNA damage and loss of telomeric sequences, whereas high TERRA also may cause DNA damage and telomere instability.

Genetic defects in this intricate machinery that maintains and protects telomeres result in excessive telomere attrition that, in humans, may translate into severe medical conditions. This review discusses the recent findings in biology, clinical presentation, management, and therapies for telomere diseases.

### Genetics of telomere diseases

Various pathogenic germline variants in genes encoding products involved in telomere maintenance may impair adequate telomere elongation and lead to multiple clinical conditions collectively called telomere diseases (telomeropathies or telomere biology disorders), molecularly characterized by short (dysfunctional) telomeres and defective telomere repair^5,17^. Excessive telomere erosion has been first implicated in dyskeratosis congenita (DC), an inherited bone marrow failure syndrome usually manifesting in the first decade of life and associated with mucocutaneous findings (nail dystrophy, leukoplakia, and skin hypopigmentation). Patients with X-linked DC were found to have lesions in the *DKC1* gene, which was later found to be part of the telomerase complex^18^. Patients had very short telomeres, and the association with marrow failure expanded the list of clinical presentations caused by defective telomerase machinery, including aplastic anemia, myelodysplastic syndrome (MDS), and acute myeloid leukemia (AML) in the hematopoietic system^5^. Subsequently, idiopathic and familial pulmonary fibrosis and liver cirrhosis (also features of DC) also were identified as telomere diseases and, in a minority of cases, found to be associated with pathogenic variants in telomere biology genes. More severe variants of DC are the Hoyeraal–Hreidarsson (accompanied by cerebellar hypoplasia, prenatal growth retardation, microcephaly, and developmental delay) and the Revesz (accompanied by exudative retinopathy) syndromes. Genetic defects in telomere-associated genes also have been identified as etiologic in Coats plus syndrome (exudative retinopathy, intracranial calcifications, and cysts).

Currently, pathogenic germline variants in at least 16 genes involved in telomere biology have been associated with telomeropathies. Of these, *TERT*, *TERC*, *DKC1*, *NHP2*, *NOP10*, *NAF1*, and *WRAP53* (*TCAB1*) compose the telomerase complex; *ACD*, *POT1*, and *TINF2* form the shelterin complex; *CTC1*, *STN1*, and *RTEL1* are associated with the adequate telomere replication; and *PARN*, *USB1*, and *ZCCHC8* play a role in RNA metabolism (Table 1)^19^.

**Table 1.** Genes involved in telomere biology mutated in telomere diseases.

Gene	Associated manifestations
*ACD*	Aplastic anemia
	Familial melanoma
	Hoyeraal–Hreidarsson syndrome
*CTC1*	Aplastic anemia
	Coats plus syndrome
	Dyskeratosis congenita
	Portal hypertension
*DKC1*	Aplastic anemia
	Cirrhosis
	Dyskeratosis congenita
	Familial interstitial pneumonia
	Hoyeraal–Hreidarsson syndrome
	Idiopathic pulmonary fibrosis
	Myelodysplastic syndrome/acute myeloid leukemia
	Solid tumors
*NAF1*	Idiopathic pulmonary fibrosis
	Myelodysplastic syndrome/acute myeloid leukemia
*NHP2*	Aplastic anemia
	Dyskeratosis congenita
	Hoyeraal–Hreidarsson syndrome
	Idiopathic pulmonary fibrosis
*NOP10*	Dyskeratosis congenita
	Idiopathic pulmonary fibrosis
*PARN*	Aplastic anemia
	Dyskeratosis congenita
	Hoyeraal–Hreidarsson syndrome
	Idiopathic pulmonary fibrosis
	Myelodysplastic syndrome/acute myeloid leukemia
*POT1*	Angiosarcoma
	Chronic lymphocytic leukemia
	Coats plus syndrome
	Familial melanoma
	Glioma
	Idiopathic pulmonary fibrosis
*RTEL1*	Aplastic anemia
	Dyskeratosis congenita
	Familial interstitial pneumonia
	Hoyeraal–Hreidarsson syndrome
	Idiopathic pulmonary fibrosis
	Myelodysplastic syndrome/acute myeloid leukemia
*STN1*	Aplastic anemia
	Coats plus syndrome
*TERC*	Aplastic anemia
	Cirrhosis
	Dyskeratosis congenita
	Hoyeraal–Hreidarsson syndrome
	Idiopathic pulmonary fibrosis
	Myelodysplastic syndrome/acute myeloid leukemia
*TERT*	Aplastic anemia
	Cirrhosis
	Dyskeratosis congenita
	Hepatopulmonary syndrome
	Hoyeraal–Hreidarsson syndrome
	Idiopathic pulmonary fibrosis
	Myelodysplastic syndrome/acute myeloid leukemia
	Melanoma
	Solid tumors
*TINF2*	Aplastic anemia
	Dyskeratosis congenita
	Hoyeraal–Hreidarsson syndrome
	Myelodysplastic syndrome/acute myeloid leukemia
	Revesz syndrome
*WRAP53*	Dyskeratosis congenita
	Hoyeraal–Hreidarsson syndrome
*ZCCHC8*	Idiopathic pulmonary fibrosis
	Myelodysplastic syndrome/acute myeloid leukemia
*USB1*	Dyskeratosis congenita

Somatic genetic rescue, which is the random occurrence of mutations in a somatic cell alleviating or annulling the effects of the pathogenic germline mutation, may occur in telomere diseases. Somatic mutations in the *TERT* promoter have been identified in hematopoietic cells, especially in older patients with pulmonary fibrosis but less frequently in marrow failure^20,21^. This *TERT* promoter mutation increases *TERT* transcription and may augment telomere elongation. This molecular event may expand hematopoietic proliferation capacity and mitigate marrow failure in some patients^21^. Additionally, back mutations, correcting the original germline pathogenic mutations, have been found in DC, improving hematopoiesis^22,23^.

Clonal hematopoiesis associated with other telomere biology genes and myeloid neoplasm-associated genes has been found in the hematopoietic tissue in telomere diseases^24,25^. Small and more significant clones have recently been observed, and the mutation pattern appears to correlate with phenotype and the affected germline gene^25^. Whereas clonal hematopoiesis is more common in patients carrying germline *TERT*, *TERC*, or *TINF2* mutations, patients with *RTEL1* or *DKC1* gene mutations are less likely to display clonal hematopoiesis. Recent findings suggest that clonal hematopoiesis is more frequent with older age and negatively impacts survival (Fernanda Gutierrez-Rodrigues, personal communication). These recent observations suggest how the pathogenic disease-causing mutations impact hematopoiesis, the affected molecular pathways, and potential advantageous mechanisms to circumvent the deleterious effects of the germline mutation. On the other hand, the appearance of driver mutations associated with myeloid neoplasms suggests the maladaptive selection of clones with a proclivity for malignant transformation.

## Clinical presentation and diagnosis

### Organ involvement

**Figure 2.**
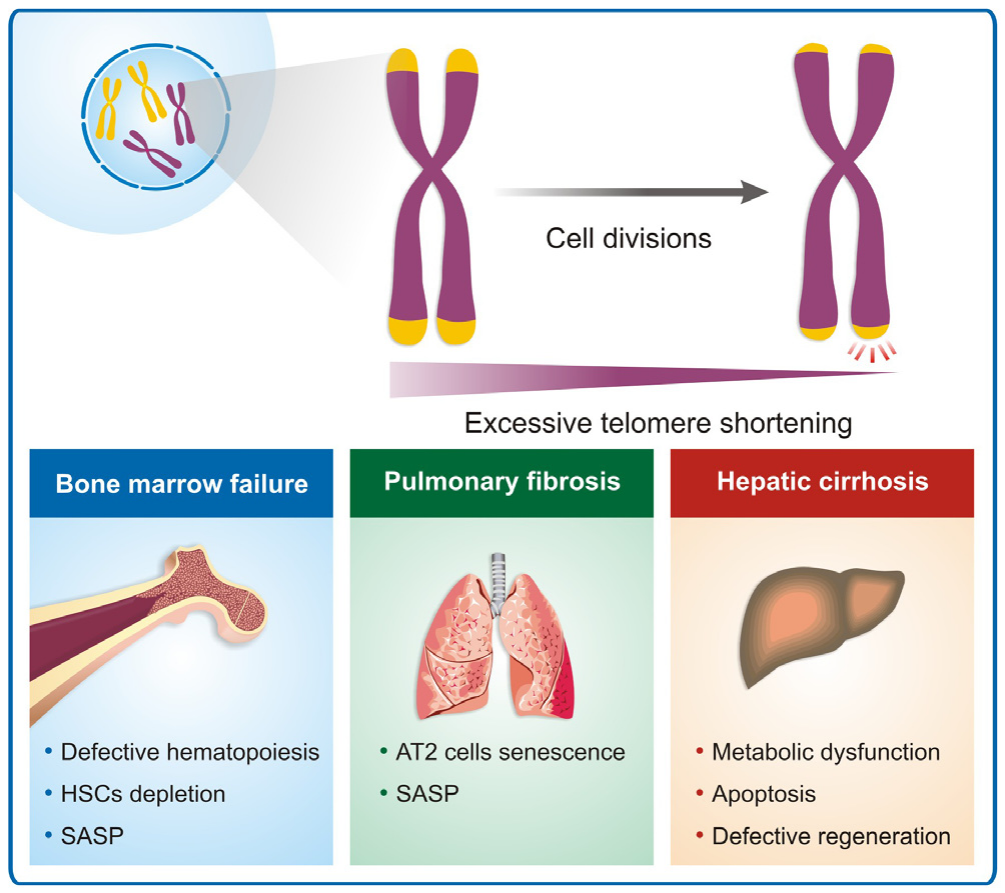
Telomere erosion and disease. Excessive telomere shortening due to genetic effects causing impaired telomere maintenance results in several clinical manifestations. In the bone marrow, telomere shortening affects the hematopoietic stem cell capacity to proliferate and genomic instability, resulting in aplastic anemia and myeloid neoplasms. In the lungs, it appears to affect the AT2 cells, inducing senescence and resulting in interstitial lung disease. In the liver, telomere attrition appears to reduce regeneration and causes metabolic dysfunction, resulting in liver cirrhosis, nodular regenerative hyperplasia, and nonalcoholic steatosis. AT2, alveolar type II epithelial; HSC, hematopoietic stem cell; SASP, senescent-associated secretory phenotype.

***Bone marrow*.** Bone marrow failure is the most common feature in patients with DC and the predominant cause of death and is a common single clinical manifestation in patients with telomere biology gene mutations, especially in the *TERT*, *TERC*, and *RTEL1* genes^5,17^. The telomerase complex is essential for the hematopoietic stem cell (HSC) pool homeostasis. Even though it is negatively regulated in quiescent cells, its activity is increased by the demand for new blood cells, inducing new progenitors to be generated^26^. When progenitors give rise to more differentiated cells, telomerase levels become undetectable. HSCs naturally lose their ability to self-renew and are highly affected with age. Defects in the telomere maintenance system lead to premature depletion of the HSC pool, causing patients to develop a hypocellular marrow (Figure 2). Consequently, patients develop cytopenias or pancytopenia. More frequently, patients seek medical assistance because of moderate aplastic anemia and may be transfusion-dependent.

The RNA component of telomerase also plays a crucial role in binding to DNA and polymerase II, recruiting the enzyme to the promoter region of myeloid genes, generating robust myelopoiesis^27^. Assays in mice reinforced the central role of TPP1 in stabilizing the shelterin complex, showing that the loss of this protein led to rapid bone marrow failure, loss of HSCs, and cell cycle arrest.

Although the molecular mechanisms triggering the decrease of HSC function under telomere attrition are not fully elucidated, cell senescence may also play an important role. First, by limiting the self-renewal capacity of the HSCs, and second, since HSCs are highly dependent on their niche, senescent cells secreting inflammatory cytokines and proteases, collectively called SASP (senescent-associated secretory phenotype), may disrupt normal hematopoiesis in these patients^28^. As mentioned above, telomere shortening also imbalances the HSC fate towards differentiation, exhausting the HSC pool^6,7^.

Telomeropathies are rare and sometimes difficult to diagnose, making treatment approaches challenging. The telomere length analysis, genetic mapping of telomere genes, and other associate phenotypes are the trivial approach for clinically diagnosing these diseases^29,30^. Epigenetic studies may help to elucidate the mechanisms causing bone marrow failure in telomeropathies, as the gain or loss of specific epigenetic marks severely affects the function of HSCs^31,32^. These combined approaches aim to anticipate diagnosis and define better treatment outcomes.

Somatic genetic rescue has been reported in individuals with germline variants in *RPA1*, *TERT*, *TERC*, *DKC1*, and *PARN*, stabilizing the telomere length and potentially ameliorating marrow failure^30^.

***Lungs*.** Pulmonary fibrosis (PF) is a clinical feature of about 20% of patients with DC, making it one of the most common manifestations in telomeric disorders^5^. The archetypical form of pulmonary fibrosis is idiopathic PF, defined by the pattern of usual interstitial pneumonia on high-resolution computed tomography or histopathology (or both), presenting characteristic patchy dense fibrosis, with remodeling of the lung architecture, peripheral reticular, and ground-glass opacity. A pattern of diffuse honeycombing is also usually observed in these patients, combined with impaired pulmonary function, indicated by low rates of forced expiratory volume, forced vital capacity, and diffusion capacity for carbon monoxide (DL_CO_)^29^. In sporadic and familial PF, many patients have short telomeres in peripheral blood leukocytes, and some of them also carry a germline pathogenic variant in a telomere biology gene^33^.

However, the exact mechanisms through which PF develops in telomere diseases remain unclear, but alveolar type II epithelial (AT2) cells appear to be protagonists^34^ (Figure 2). AT2 cells are the progenitors of the alveolar epithelium, also responsible for maintaining the microenvironment of the alveolus by providing pulmonary surfactant, collectins, and a variety of anti-inflammatory and antimicrobial substances^35^. For instance, silencing the coding gene for the shelterin component TRF1 in type I collagen-expressing fibroblasts causes lung edema but not fibrosis in mice, whereas silencing the same gene in AT2 cells leads to fibrosis, with alveolar septal thickening, architectural distortion, and deposition of interstitial collagen and observable honeycombing pattern in radiological images^36^. In PF patients harboring *TERT* mutations, AT2 cells present the shortest telomere length and the highest γH2AX signal when compared with club cells and myofibroblasts^33,37^.

Telomere attrition in lung cells also contributes to the production of SASP, constituted by various interleukins, chemokines, growth factors, and proteases^38^. When SASP is secreted, neighboring cells are triggered to enter a senescent state, a phenomenon called paracrine senescence. In PF, AT2 cells are likely the initiators of SASP, originating in a pro-fibrotic environment within the lungs because of the decrease in proliferative capacity, inability to repair tissue damages, and the production of collagen by myofibroblasts in response to tumor growth factor beta (TGF-β)^39^.

***Liver*.** Telomere diseases are associated with liver cirrhosis, nonalcoholic fatty liver disease, hepatic nodular regenerative hyperplasia, and hepatocellular carcinoma, which may occur in about 7% of all patients with DC^5^ (Figure 2). When considering telomere diseases other than DC, recent studies show that liver involvement may occur in a higher percentage of patients. In a cohort of 121 individuals with telomeropathies and variants in *TERT*, *TERC*, and *TINF2*, 40% were found to have some extent of liver disorder. The significant findings were elevated hepatic enzymes, increased liver echogenicity on ultrasound, hepatomegaly, portal hypertension, and nodular lesions^40^.

There is an increased incidence of *TERT* and *TERC* variants in patients with cirrhosis and hepatocellular carcinoma^41–43^. More recently, *RTEL1*, *TERT*, *NHP2*, and *TINF2* variants have also been found in patients with end-stage cirrhosis waiting for liver transplantation^44^. The mechanisms linking telomere shortening to cirrhosis development are still unclear. Cellular senescence is thought to play a role, especially in the case of chronic liver injury in which regeneration is impaired. However, the reasons for a pro-fibrotic response and hepatic stellate cell activation are not clear^45^. Therapies for telomeropathies may contribute to the liver insult. HSC transplant has the potential to cause hepatic damage due to transplant regimen toxicity; multiple red blood cell transfusions also result in iron overload^46^.

Defective lipid metabolism also is observed when telomerase is not functional, as metabolic changes in *Tert* “knockout” (*Tert^−/−^*) but not *Terc* “knockout” (*Terc^−/−^*) mice fed with a high-fat diet have been described^47^. The absence of the TERT enzyme was associated with increased lipid deposition in the liver and higher levels of serum glucose, cholesterol, alanine transaminase (ALT), and hepatic triglycerides, resulting in clinical liver steatosis, severe hepatocyte injury, and broad metabolic dysfunction.

Moreover, telomerase dysfunction in *Terc^−/−^* and *Tert^−/−^* mice activates p53-dependent mechanisms that regulate mitochondrial physiology and metabolism, causing impaired mitochondrial function, such as reduction in oxygen consumption, defective electron transport chain activity, and diminished ATP synthesis^48^. Particularly in the liver, p53 activated due to telomere shortening also represses both cytoplasmic and mitochondrial sirtuins, a class of NAD(+)-dependent enzymes essential for cellular metabolism, transcriptional silencing, post-translational modifications, and DNA repair. In addition, in *Tert^−/−^* mice treated with carbon tetrachloride (CCl_4_), supplementation with NAD(+) precursors, known to increase sirtuins levels, alleviated the hepatic fibrosis development and protected telomeres from attrition, in comparison with the animals that did not receive NAD(+) supplementation^49^.

Altogether, these data suggest that successive hits and impaired regeneration are the underlying factors of hepatic commitment in short-telomere syndromes. In cryptogenic cirrhosis or idiopathic portal hypertension, telomeropathies should be investigated as a possible cause, especially if there is a family history of liver disease or other short telomere–related manifestations.

The organs involved in a given patient depend on genetic, epigenetic, and environmental factors. The affected gene or specific mutation alone does not necessarily determine the phenotype. In fact, in one family with a given gene mutation (e.g., *TERT*), one individual may develop aplastic anemia and the other pulmonary fibrosis. A third one may present marrow failure and cirrhosis, and others may be asymptomatic. However, some factors may drive the phenotype more prominently. When the affected gene is X-linked (*DKC1*) or autosomal recessive (e.g., *RTEL1*), patients usually have very short telomeres from an early age and a more severe phenotype (DC) early in life (first decades of life). Autosomal dominant mutations in *TINF2* also cause very short telomeres and more severe manifestations. On the other hand, heterozygous mutations (*TERT*, *TERC*, and *RTEL1*) causing haploinsufficiency are associated with less acute telomere erosion, and clinical signs and symptoms may appear later in life. Marrow failure is a characteristic phenotype in children with DC but also is the single clinical feature in young adults as the result of HSC exhaustion^5^. Fibrotic diseases, especially pulmonary fibrosis, are associated with aging and usually appear after age 60 as a consequence of heterozygous mutations and less acute telomere attrition. Also, somatic genetic rescue in the hematopoietic compartment is more common in older patients with pulmonary fibrosis^20,21^. One hypothesis is that somatic mutations may rescue, at least in part, failed hematopoiesis, avoiding aplastic anemia earlier in life and allowing patients to reach a longer life span and eventually develop lung disease. Finally, environmental factors, such as smoking, alcohol, and viral infections, may modulate the phenotypes and organ involvement.

## How to diagnose telomere diseases

Traditionally, a telomere disease is suspected in patients with bone marrow failure associated with physical anomalies suggestive of DC (nail dystrophy, leukoplakia, skin hyperpigmentation, and early graying of hair) or a positive family history of marrow failure, MDS, AML, or lung or liver disease or a long history of moderate aplastic anemia or macrocytic anemia and a hypoplastic marrow. Also, patients with myeloid neoplasms (MDS and AML) with a positive family history should be considered for telomeropathy screening.

Today, a much wider group of patients should be suspected of telomere disease. Patients with sporadic or familial idiopathic pulmonary fibrosis are suspicious of a telomeropathy, especially with extra-pulmonary manifestations, such as cryptogenic liver cirrhosis, macrocytosis, thrombocytopenia, marrow failure, skin changes (early gray hair), or family history of lung, liver, or marrow disease. Similarly, patients with liver disease associated with marrow failure and/or lung interstitial disease, skin changes, or family history should be considered for screening.

Once a telomere disease is suspected, patients should be tested for telomere length. Although several methods are available, flow-FISH (flow cytometry combined with fluorescent *in vitro* hybridization) appears to be more accurate^50^. Leukocyte telomere length is usually expressed in kilobases, but its interpretation should consider the patient’s age because of physiologic telomere loss. Telomeres are longest at birth and shorten at 40 to 60 base pairs per year. Telomere length below the tenth percentile for age is defined as “short,” and telomeres below the first percentile are considered “very short.”

If telomeres are short or very short by flow-FISH (or if the patient history is very suspicious of a telomere disease), the patient should be considered for genetic testing for pathogenic variants in genes involved in telomere maintenance (Table 1). Mutations may be bi-allelic or tri-allelic (involving more than one gene locus), especially in DC. Still, in most cases of aplastic anemia and pulmonary fibrosis, only one allele is affected (autosomal dominant inheritance). Appropriate interpretation of variants is crucial in diagnosis, especially when massive parallel sequencing is applied, and *in silico* and functional studies may be necessary for interpretation. Guidelines by the American Society of Medical Genetics should be followed^51^.

When genetic testing is performed, genetic counseling is paramount, as the inheritance pattern is multiple depending on the gene, and penetrance is variable. Family members who are potential donors for HSC transplant must be screened for telomere length and mutations before transplantation. Another important issue for the diagnosis of telomere disease and genetic counseling is the somatic genetic rescue when a mutation occurs in somatic cells mitigating or abolishing the effects of the pathogenic mutation^52^. Genetic somatic rescue may obfuscate the germline pathogenic variant in routine genetic sequencing, and clinicians and geneticists should be aware of a “cryptic” pathogenic mutation^53^. Additionally, a recent retrospective study suggests that *TERT* promoter somatic mutations may impact hematologic complications after lung transplant with better outcomes^54^. Somatic genetic rescue also appears to influence malignant transformation in the hematopoietic compartment^55^.

Identifying a patient with a telomere disease among those with bone marrow failure, pulmonary fibrosis, or liver disease has several clinical implications. First, it guides therapeutic options. For instance, telomeropathy patients with aplastic anemia do not benefit from immunosuppressive therapy, otherwise indicated for patients with immune aplastic anemia. In addition, bone marrow transplant conditioning regimens have to consider the genetic status, as telomeropathy patients are more sensitive to ionizing radiation and chemotherapy. Similar therapeutic approaches apply to patients with pulmonary fibrosis. For instance, telomeropathy patients with lung involvement are less tolerant to immunosuppressants^56^. Second, the identification of a telomeropathy has prognostic repercussions. Patients with pulmonary fibrosis and a telomere biology gene variant have worse overall survival in comparison with those without a mutation^57^. Additionally, the identification of a telomeropathy implies careful management and follow-up of other organs and systems that may be affected and directs screening for cancer susceptibility^58^. Finally, the discovery of a genetic etiology may have consequences for other family members, and genetic counseling is preeminent.

## Therapies for telomere diseases

Severe aplastic anemia due to telomere maintenance defect may be cured by HSC transplantation. However, pulmonary and hepatic complications have been historically high because of conditioning regimen toxicity, limiting transplant indication. In the last decade, novel reduced-intensity regimens avoiding cytotoxic chemotherapy and comprehensive donor matching have significantly improved outcomes with encouraging results^59^. Usually, patients with severe cytopenias (severe neutropenia or transfusion dependence) and a high risk of malignant evolution are indications for HSC transplantation. It is important to emphasize that, given the complexity and rarity of these cases, patients with telomere diseases should be transplanted in referral expert centers.

Likewise, a lung transplant is the only definitive therapy and may be attempted for interstitial lung disease, but indication also is limited because of concomitant cytopenias and liver involvement. Patients with telomere disease display faster disease progression and more limited transplant-free survival in comparison with other causes of interstitial lung disease^56,57^. Additionally, telomeropathy patients appear to have higher post-transplant mortality and chronic lung allograft dysfunction^18,47^. Additionally, telomeropathy patients may not adequately tolerate transplant-related immunosuppression with increased hematologic and renal toxicities^60^. There is no specific standard recommendation for treating lung disease in telomeropathy patients, given the lack of data. However, patients should be treated according to the standard guidelines for their disease (e.g., antifibrotics)^61^.

There is limited information on the outcomes of liver transplantation for patients with telomere and chronic liver disease. A collaborative retrospective study is being conducted; thus far, 21 patients with telomere disease were transplanted for severe liver disease with encouraging results (Michele Wang, personal communication). Liver transplantation may be indicated because of chronic liver failure with severe liver dysfunction or complications of liver involvement (portal hypertension, hepatopulmonary syndrome).

Male hormones have been used to treat cytopenias in inherited bone marrow failure syndromes, including telomeropathies, in heterogeneous results. In telomere diseases, at least two prospective studies demonstrated the benefit of androgen therapy on (a) telomere elongation and (b) hematologic recovery. One trial using danazol at 800 mg daily for two years demonstrated telomere elongation during therapy associated with hematologic recovery in 79% of patients and transfusion independence. Low-grade adverse events occurred in about one third of patients^62^. We treated 17 patients with telomeropathy with nandrolone decanoate 5 mg/kg every other week for two years. Nandrolone treatment was also associated with telomere elongation and hematologic response in 63% of patients^63^. Nandrolone had no impact on the dynamics of clonal hematopoiesis bearing driver mutations associated with myeloid neoplasms. Additionally, the pulmonary function assessed by the diffusing capacity for carbon monoxide appeared to be stable during nandrolone therapy. However, the number of evaluable patients was too small to draw firm conclusions.

Other pathways have been explored *in vitro* as a potential target for therapies. PAPD5 is a noncanonical poly(A) polymerase that destabilizes TERC, the telomerase RNA component. PAPD5 silencing in patient-derived embryonic cells rescues TERC, restores telomerase activity, elongates telomeres, and improves definitive hematopoietic differentiation. Small molecules that are PAPD5 inhibitors also recover telomerase activity and elongate telomeres in patient-derived induced pluripotent stem cells^64,65^. These findings suggest that novel therapeutic approaches using small molecules may become available to restore telomerase activity in telomere elongation in patients with telomere diseases. It is not clear, however, whether this approach will be effective for the different germline genetic lesions present in telomeropathies or restricted to those in which telomerase itself is defective.

## Conclusions

The concept of telomere diseases has linked clinically distinct disorders (aplastic anemia, pulmonary fibrosis, and liver cirrhosis) based on the shared molecular mechanism: severe telomere erosion. This understanding is fundamental for appropriate diagnosis and therapy. We still do not understand clearly how telomere erosion results in fibrotic disorders in the lungs and liver, which prevents the development of novel therapies for these conditions as well as preventive strategies. The intricate genomic architecture arising in the hematopoietic tissue based on somatic genetic rescue indicates, along with androgen effects on hematopoiesis, that telomerase modulation may ameliorate marrow failure in patients.
